# Supplementary material for: Prevalence and correlates of diagnosed and undiagnosed epilepsy and migraine headache among people with severe psychiatric disorders in Ethiopia
Source: PLoS One. 2020 Nov 20;15(11):e0241581. doi: 10.1371/journal.pone.0241581 (PMC7678996; doi:10.1371/journal.pone.0241581)
Supplement: Attachment — Submitted filename: PLOS ONE_Reviewers Comments_1.doc [file pone.0241581.s002.doc]

The epidemiology of diagnosed and undiagnosed chronic neurological disorders with episodic manifestations in People with Severe Psychiatric Disorders in Ethiopia

Ayano G ..

I find this to a very interesting paper and see no major concern. But, I would like the following points revised.

1. This paper mainly focussed on co-morbid epilepsy and migraine headache. But, the way it is presented in the title, described in the texts and discussed makes it look like, the focus is many, if not all neurological disorders. Taking this as “Epidemiology of…” also takes this work out of its context. I would suggest this is revisited. “Diagnosed and undiagnosed epilepsy and migraine headache co-morbidity in people with Severe Psychiatric Disorders in Ethiopia” or so.
2. This is a cross-sectional study where researchers assessed patients or interviewed. My understanding is that all patients were interviewed and I did not see why they looked into past diagnoses. Is this a life time prevalence data? They may need to comment on why the did that.
3. Readers may like to know more about how they used the instruments. Was it translated, validated? This includes ILAE and ICHD-3. Was this self reported on interview based. Please give some comments.
4. There were only 3/309 SPD patients who had undiagnosed epilepsy and 12/309 SPD patients with undiagnosed migraine headache. The way it looks in the abstract and texts is a bit exaggerated as 60% and 100%. I suggest that is made clear: 2/3 (60 percent) of co-morbid epilepsy and 12/12 (100 per cent) of co-morbid migraine headache were undiagnosed.
