# Supplementary material for: Prevalence and correlates of diagnosed and undiagnosed epilepsy and migraine headache among people with severe psychiatric disorders in Ethiopia
Source: PLoS One. 2020 Nov 20;15(11):e0241581. doi: 10.1371/journal.pone.0241581 (PMC7678996; doi:10.1371/journal.pone.0241581)
Supplement: Attachment — Submitted filename: Comments to PLOSONE.docx [file pone.0241581.s003.docx]

**Comments**

**Sample size calculation**

On sample size calculation, it is not clear that there are many comorbidities with severe psychiatric disorder. Therefore, better to deal with the prevalence of epilepsy and migraine headache. Report that you have considered the maximum sample size.

On statistical analysis

The researcher mentioned the independent variables; just remove from the statistical analysis.

**Result**

Sociodemographic characteristics of the participants

Rather than mentioning as the following “Sociodemographic and clinical data for our participants were summarized in Table 1” it is better to write (table 1) at the end of the paragraph.

The prevalence of undiagnosed neurological disorders in patients with SPD

Don’t put table 2 in each paragraph. Just put it at the end of last paragraph.

The prevalence and associated factors of neurological disorders in people with SPD

Discussion

The researchers should at least search for additional literature that has been done from different data bases. If no they can compare their finding which is cross-sectional study with the meta-analysis.

Some of the justification for the difference in the finding are not scientifically sound according to the context that they are studying. Which the stating as follows

“There are wide ranges of explanations for higher prevalence rates of epilepsy among people with severe psychiatric disorders as compared with the reported prevalence in the general population. One of the possible explanations is that these disorders might have shared genetic factors”

Therefore, better to explain in more acceptable and in details so that the reader will be convinced from the finding.

**References**

Outdates references should be corrected by the recently published works. I encourage the researchers even to look into additional references from Ethiopia.

**General comment**

The manuscript is scientifically sound and well written
